# Supplementary material for: Biomimetic Gradient Hydrogels with High Toughness and Antibacterial Properties
Source: Gels. 2023 Dec 21;10(1):6. doi: 10.3390/gels10010006 (PMC10815424; doi:10.3390/gels10010006)
Supplement: Supplementary file 1 [file gels-10-00006-s001.zip › gels-2739050-supplementary.pdf]

Supporting Information

Biomimetic Gradient Hydrogels with High Toughness and Antibacterial Properties

Mingzhu Zeng[1,2,3], Zhimao Huang[2,3], Xiao Cen[2,3], Yinyu Zhao[2,3], Fei Xu[1], Jiru Miao[2,3], Quan Zhang[1,*], Rong Wang[2,3,*]

[1] Institute of Smart Biomedical Materials, School of Materials Science and Engineering, Zhejiang Sci-Tech University, Hangzhou 310018, China

[2] Zhejiang International Scientific and Technological Cooperative Base of Biomedical Materials and Technology, Institute of Biomedical Engineering, Ningbo Institute of Materials Technology and Engineering, Chinese Academy of Sciences, Ningbo, 315201, China

[3] Ningbo Cixi Institute of Biomedical Engineering, Ningbo 315300, China

*Correspondence address. Tel: 0571-86840651; E-mail: quanzhang@zstu.edu.cn (Q. Zhang); rong.wang@nimte.ac.cn (R.Wang).

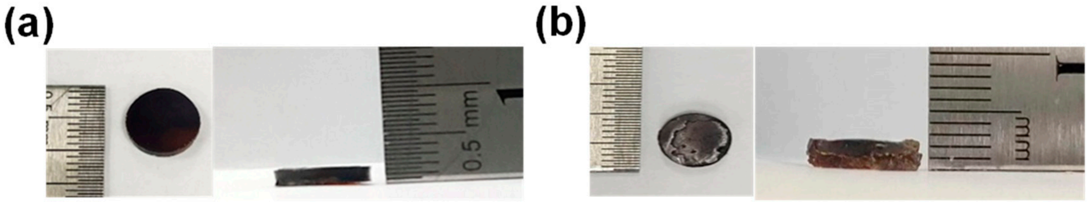

Figure S1. Photos of TAPS-Cu15 hydrogel (a) before compression and (b) after compression at 90% strain.

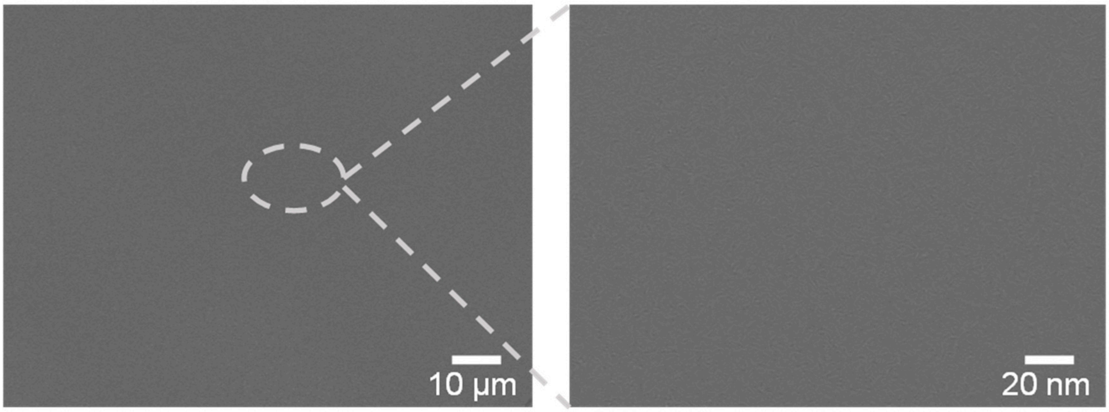

Figure S2. SEM images of cross-sections of TAPS-Cu15 hydrogel.
